# Design of a Sensor-Technology-Augmented Gait and Balance Monitoring System for Community-Dwelling Older Adults in Hong Kong: A Pilot Feasibility Study

**DOI:** 10.3390/s23188008

**Published:** 2023-09-21

**Authors:** Yang Zhao, Lisha Yu, Xiaomao Fan, Marco Y. C. Pang, Kwok-Leung Tsui, Hailiang Wang

**Affiliations:** 1 School of Public Health (Shenzhen), Sun Yat-sen University, Shenzhen 518000, China; zhaoy393@mail.sysu.edu.cn; 2 School of Design, The Hong Kong Polytechnic University, Hong Kong, China; lisha33.yu@polyu.edu.hk; 3 College of Big Data and Internet, Shenzhen Technology University, Shenzhen 518000, China; fanxiaomao@sztu.edu.cn; 4 Department of Rehabilitation Sciences, The Hong Kong Polytechnic University, Hong Kong, China; marco.pang@polyu.edu.hk; 5 Grado Department of Industrial and Systems Engineering, Virginia Polytechnic Institute and State University, Blacksburg, VA 24061, USA; kltsui@vt.edu

**Keywords:** gait and balance, sensor technology, prediction, feasibility

## Abstract

Routine assessments of gait and balance have been recognized as an effective approach for preventing falls by issuing early warnings and implementing appropriate interventions. However, current limited public healthcare resources cannot meet the demand for continuous monitoring of deteriorations in gait and balance. The objective of this study was to develop and evaluate the feasibility of a prototype surrogate system driven by sensor technology and multi-sourced heterogeneous data analytics, for gait and balance assessment and monitoring. The system was designed to analyze users’ multi-mode data streams collected via inertial sensors and a depth camera while performing a 3-m timed up and go test, a five-times-sit-to-stand test, and a Romberg test, for predicting scores on clinical measurements by physiotherapists. Generalized regression of sensor data was conducted to build prediction models for gait and balance estimations. Demographic correlations with user acceptance behaviors were analyzed using ordinal logistic regression. Forty-four older adults (38 females) were recruited in this pilot study (mean age = 78.5 years, standard deviation [SD] = 6.2 years). The participants perceived that using the system for their gait and balance monitoring was a good idea (mean = 5.45, SD = 0.76) and easy (mean = 4.95, SD = 1.09), and that the system is useful in improving their health (mean = 5.32, SD = 0.83), is trustworthy (mean = 5.04, SD = 0.88), and has a good fit between task and technology (mean = 4.97, SD = 0.84). In general, the participants showed a positive intention to use the proposed system in their gait and balance management (mean = 5.22, SD = 1.10). Demographic correlations with user acceptance are discussed. This study provides preliminary evidence supporting the feasibility of using a sensor-technology-augmented system to manage the gait and balance of community-dwelling older adults. The intervention is validated as being acceptable, viable, and valuable.

## 1. Introduction

Falls have been cited as an important issue leading to injury, morbidity, and mortality among older adults worldwide. Among the various driving factors of falls, gait and balance impairment has been validated as a critical causal factor [1]. The routine assessment of gait and balance can effectively reduce and prevent falls via anomaly detection, timely warnings, and appropriate intervention [2]. Nowadays, community-dwelling older adults usually undergo gait and balance evaluations by physiotherapists using scoring scales, such as the Berg balance scale (BBS) [3], the 3-m timed up and go (3M-TUG) test [4], the five-times-sit-to-stand (FTSTS) test [5], the Romberg test [6], and the balance evaluation systems test (BESTest) [7]. However, the routine assessment of gait and balance requires extensive healthcare resources. Additionally, such assessment includes a time-consuming and challenging subjective analysis of the patient’s mobility status. By the beginning of 2022, the number of residents aged 65 years or older in Hong Kong was approximately 1.43 million, while there were only 3918 registered physiotherapists [8]. Such limited professional resources cannot sufficiently enable the timely detection of the deterioration of gait and balance behaviors. This situation may even worsen as the older adult population continues to grow. Moreover, although conventional assessments allow comprehensive quantitative comparisons of performance in various tasks, their accuracy relies heavily on the subjective judgment of the investigator (i.e., physiotherapist) and an assessment may not fully reflect the older person’s actual situation. Furthermore, clinical time constraints and a lack of technology-augmented assessments would prevent healthcare professionals from performing evaluations periodically [9]. In an attempt to remedy this, there is a crucial need for an intelligent gait and balance monitoring system, which requires the least involvement from specialists (e.g., physiotherapists), for older adults and caregivers in the community. Such a system is expected to enable the early detection of anomalies in gait and balance behaviors with credible sensitivity and specificity using sufficient quantitative information.

Big data analytics integrated with advanced sensing technologies could be a solution to meeting the urgent need. Sensors can efficiently capture motion-related data from real-world environments where older adults perform daily activities, while statistical learning methods can process the collected data for meaningful outcomes [9,10]. The inertial measurement unit (IMU), which typically comprises an accelerometer, a gyroscope, and a magnetometer, has been widely used for gait and balance assessment owing to its low cost, high efficiency, small size, easy implementation, and ability to record movement signals [1,11]. However, a single IMU can only capture partial information about human activities, whereas wearing multiple IMUs can result in a poor wearing experience. In contrast, depth cameras (e.g., the Microsoft Kinect, Microsoft, Redmond, WA, USA) are effective tools for the three-dimensional reconstruction of human activities. The built-in artificial intelligence (AI) algorithms provide effective access to skeletal data, prompting researchers to use depth cameras for gait and balance assessments [12,13]. However, depth cameras have limitations in recognizing some movements, such as turning, where the data from the left and right sides of bodies overlap, and skeleton data cannot be fully tracked. Using an IMU in conjunction with a depth camera merits further investigation in gait and balance research.

Therefore, in the present study, we designed a surrogate monitoring system based on sensing technology, multi-sourced data stream analytics, and statistical monitoring to assist the professional assessment of gait and balance among older adults. Specifically, we explored the integration of an IMU and depth camera to depict body movements. The derived datasets would be useful for the quantitative, objective, and unobtrusive assessment of the functional gait and balance in older adults during the performance of clinical standard functional tasks. We also explored the feasibility of a prototype system for community-dwelling older adults in Hong Kong to evaluate whether such a system could be used among the target population.

## 2. Methods

### 2.1. Design of the System Architecture

**Figure 1:**
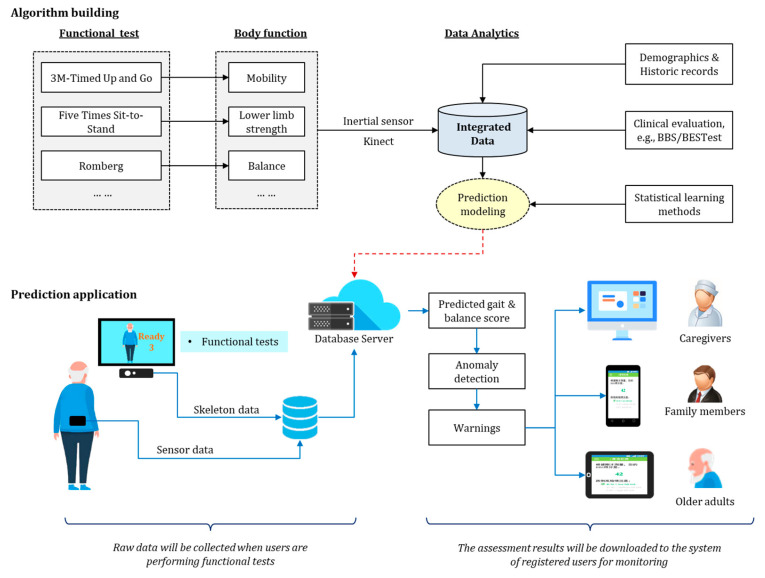
Schematic diagram of the proposed system architecture.

Figure 1 presents the system architecture having two main developmental stages: algorithm building and prediction application. In the algorithm building stage, raw signal data were collected via an IMU and depth camera while each participant was performing various clinical measurement tests, and the data were then transferred to a data analysis module via Bluetooth in real time. Additionally, the database hosted on a remote server managed the related personalized health records, together with the gait and balance scales provided by physiotherapists. Statistical learning models were fitted to correlate the personalized sensor and non-sensor data with gait and balance scales. In the prediction stage, well-trained learning models were applied to the newly captured data, and the predicted values of the gait and balance scales were then output. The system continuously evaluated the fall risk level by monitoring gait and balance, and it generated a warning alarm once the inferred fall risk exceeded a normality threshold.

Users were first asked to provide personal demographic information and historic records (e.g., age, gender, and fall history). They were then instructed to wear an IMU and perform the selected standard functional tasks in front of a depth camera so that sensor data could be correctly recorded. After the functional tasks, the users’ gait and balance were evaluated by registered physiotherapists (PTs) using selected clinical scoring tools. These three types of collected data were fed into a database repository for subsequent data validation, data integration, and model building. Subsequently, based on the well-trained prediction models, the calibrated algorithms were integrated into a system that would be used for the longitudinal monitoring of gait and balance in older adults. The proposed system prototype would be capable of sending warnings about abnormal behaviors and non-clinical advice on improvements to users through mobile devices.

We selected three standard functional tasks as tests for raw data collection and two golden standard tests for gait and balance evaluation.

- Task-1: 3-m timed up and go (3M-TUG) test

Users were instructed to stand up from a chair, walk a distance of 3 m at a natural pace while ensuring safety, then turn around, walk back, and sit down into the same chair. The 3M-TUG test has been recognized as a routine screening test with high reliability and validity for falls. Its intratester and intertester reliability have been reported as high in elderly populations (Intraclass correlation coefficient (ICC) = 0.92 − 0.99) [14]. For identifying people who fall, the TUG was found to have sensitivity and specificity of 89% [15]. A longer 3M-TUG time indicates lower mobility of the user and a higher risk of falling [16,17].

- Task-2: Five-times-sit-to-stand (FTSTS) test

Users were instructed to stand up and sit down as quickly as possible five times, with arms folded across their chest. The FTSTS test has been used to examine lower extremity strength and to determine an individual’s risk of falling [5,18] with moderate to excellent test–retest reliability (ICC = 0.64 − 0.96, mean ICC = 0.82), even after adjusting for a history of falls [5].

- Task 3: Romberg test

Users were instructed to stand with their two feet together and their arms held next to the body, first standing quietly with eyes open and subsequently with eyes closed. The Romberg test has been used to diagnose sensory ataxia, a gait disturbance caused by abnormal proprioception, to measure the degree of standing postural sway (e.g., the center of pressure) [6,19,20]. Specially, the ICC value quantifies the reliability of the Romberg test as excellent in individuals with Parkinson’s disease [21].

- Gait and balance evaluation-1: BBS

Given its high reliability in older adults [22,23], the BBS was used to evaluate the individuals’ balance performance on 14 specific functional tasks, with a score ranging from 0 to 56 points and a higher score indicating better performance [24].

- Gait and balance evaluation-2: Brief-BESTest (Balance evaluation systems test)

The brief-BESTest, a short version of the BESTest [25], was conducted to evaluate the individuals’ performance in six subsections, namely, biomechanical constraints, stability limits and verticality, anticipatory postural adjustments, postural responses to external perturbations, sensory orientation during stance, and stability of the gait [7]. The brief-BEST score ranges from 0 to 24 points, with a higher score indicating better performance.

### 2.2. Feature Extraction and Prediction Models

A set of features extracted from both IMU and Kinect camera data collected during the standard functional tests was used as input for the predictive model, and the numerical BBS and BESTest scores were taken as the model output. In the case of the IMU data, the features for modeling obtained from different combinations of functional tasks, sensor placements, and feature categories varied greatly [26]. Therefore, when selecting significant features in our study, we applied different inclusion criteria to each of the segmented phases according to data availability [26,27,28,29,30,31,32]. Specifically, for gait data, a feature was selected if it met the criteria that [the feature was reported significant in at least two studies (*p* < 0.05)] AND [the feature was computed for a walking task] AND [the feature was independent of sensor placement and type (e.g., the number of steps)]. For postural action data, a feature was selected if it was statistically significant (*p* < 0.05), regardless of the participant’s pathological condition and sensor placement. The features extracted from the IMU data can be categorized further according to linear acceleration/angles, spatial-temporal distribution, and frequency [27]. For the Kinect key-point data, a set of timing- and speed-related measurements can be derived as features [33]. More information related to innovative methodologies for IMU-based gait analysis can be found in [28], and for gait and postural assessment using the Kinect camera can be found in [34]. Subsequently, all of the features extracted from both the IMU and Kinect data were used as model inputs.

In the next step, we used three regularized regressions—ridge, lasso, and elastic net regressions—to predict the BBS and BESTest scores. Regularized regression models were selected according to the effectiveness of their predictability and interpretability in the literature. The use of a ridge penalty is known to reduce the coefficients of correlation of predictors [35], whereas lasso regression tends to select one predictor while discarding the others [36]. The use of the elastic net penalty mixes these two concepts, with the objective function taking the form of a loss and penalty:

(1) $$arg\;\underset{\beta }{\min}{\left\|y-X\beta \right\|}_{2}^{2},\;s.t.\left(1-\alpha \right)/2{\left\|\beta \right\|}_{2}^{2}+\alpha {\left\|\beta \right\|}_{1}^{\;}\leq t,$$

where α is the elastic net penalty that controls the balance between the ridge and lasso regressions, ${\left\|\beta \right\|}_{2}^{2}=\sum_{j=1}^{p}\beta_{j}^{2}$ is the L2-norm of β, ${\left\|\beta \right\|}_{1}=\sum_{j=1}^{p}\left|\beta_{j}\right|$ is the L1-norm of β, and t is a tuning parameter. The elastic net regression reduces to a simple ridge regression when α=0 and to a lasso regression when α=1.

The mean absolute error and root mean square were used to measure the accuracy of the predicted BBS and BESTest scores. The data collected in the present study would also be analyzed by adopting a 10-fold cross validation approach. Additionally, the correlations between the BBS and BEST scores predicted using our models and the actual BBS and BEST scores determined by registered PTs would be tested in practice.

### 2.3. Testing Protocol

A pilot study was conducted with three elderly care centers affiliated with a local non-government organization providing community services to older adults [37]. The purpose of the pilot study was to collect raw data for developing the system algorithm and to examine the feasibility of the proposed system among community-dwelling older adults.

#### 2.3.1. Participants

We recruited older adults who met all of the inclusion criteria of being at least 65 years old, living in a community setting, having the ability to walk independently or with a walking aid, having normal (or corrected-to-normal) vision, and having the ability to provide informed consent. We excluded older adults who had abnormal vision, disability of walking, and/or life-threatening illnesses, as they would likely be unable to complete the gait and balance assessment. Each participant was given a 50 HKD supermarket coupon as a token of appreciation for completing the study. The pilot study was approved by the Research Ethics Committee of the affiliated university of the authors (reference number: 3-2020-02-F). All participants provided written informed consent before the initiation of the study.

#### 2.3.2. Data Collection

After recruiting eligible participants from the local care centers for older adults, trained research assistants (RAs) visited the centers to collect written informed consent and background data, including age, gender, body weight, stature, chronic disease history, fall history, fear of falling using the activities-specific balance confidence (ABC) scale [38], mental health condition using the Montreal cognitive assessment (MoCA) test [39], and a health index using a score between 1 and 5 points representing the health status from poor to excellent [40]. During the visit, each participant was required to first put on an elastic belt with a commercial IMU (Wit-motion JY901B, Shenzhen, China; including an accelerometer and a gyroscope with three axes, 16-bit resolution, a sampling frequency of 40 Hz, and a built-in Kalman filter) located on the L4 vertebra of the participant’s back, and then to complete the three standard functional tests in front of a Microsoft Kinect camera, with the distance ranging from 1 m to 4 m. Following the performance of the tasks, a PT and a trained RA measured the participants’ BBS and BESTest scores, respectively, according to standard procedures [24,41]. Subsequently, each participant was required to complete a customized questionnaire on the feasibility of the system. The questionnaire was designed based on validated scales from an extensive literature review of studies on geriatric technology acceptance. Modifications were made for some measurement items to meet the context of our proposed system. The questionnaire included a brief description of the gait and balance system to assist understanding, followed by the items measuring the users’ perceptions of the system. A 7-point Likert-type scale, ranging from 1 (strongly disagree) to 7 (strongly agree), was adopted for ranking the subjective evaluation in terms of the perceived usefulness (e.g., “using the gait and balance system helps me save time in managing my health”) [42], perceived ease of use (e.g., “learning to use the gait and balance system is easy for me”) [42], attitude (e.g., “using the gait and balance system is a good idea”) [42], task–technology fit (e.g., “using the gait and balance system fits with my health management requirement”) [43], trust (e.g., “the gait and balance system is reliable”) [44], and intention to use (e.g., “I intend to choose this system when I need it in the future”) [45].

#### 2.3.3. Data Summary

Descriptive statistics were calculated for demographic variables, gait and balance performance, and perceptions of the proposed system. Ordinal logistic regression was conducted to assess the demographic correlation with the perceived acceptability, with calculations of odds ratios and 95% confidence intervals. The analysis was performed using SPSS 24.0 (IBM, Armonk, NY, USA). The significance level was set at 0.05.

## 3. Results and Discussion

The present study aimed to develop and evaluate the feasibility of a sensor-technology-augmented gait and balance monitoring system for older adults. The proposed system is based on predicting BBS scores and BESTest scores from the multi-sourced data stream gathered with an IMU and a Kinect depth camera during the execution of three standard functional tests.

### 3.1. Demographics

Forty-four older adults (mean age = 78.5 years, standard deviation [SD] = 6.2 years; 38 females) completed the study. Table 1 presents their demographic information and health-related assessments. According to the Health Index results, approximately 63.6% of the participants (*n* = 28) perceived their health as good or excellent. Approximately 79.5% of the participants (*n* = 35) had mild cognitive impairment, with a score of 26 taken as a cut-off value for the diagnosis of mild cognitive impairment in MoCA [46]. Approximately 56.8% of the participants (*n* = 25) had a fear of falling in performing daily activities, in that their ABC values were lower than 67%, a cut-off value for fall prediction [47]. Approximately 61.4% of the participants (*n* = 27) had brief-BESTest scores lower than the cut-off of 15.6 [22], and 70.5% (*n* = 31) had BBS scores lower than the cut-off of 52.5 [22], which are cut-offs for the presence of a fall risk.

### 3.2. Feasibility

**Figure 2:**
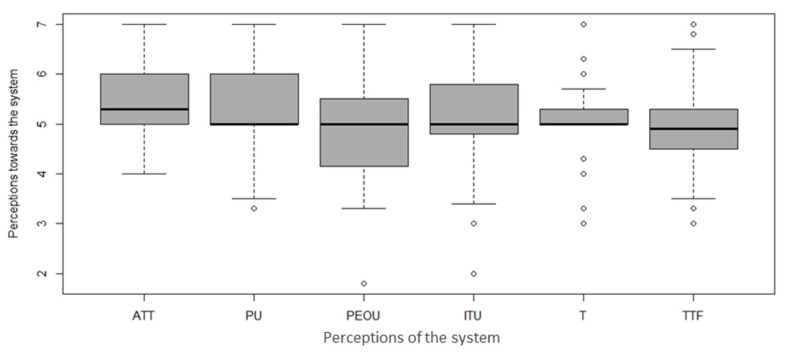
Boxplots of the participants’ perceptions of the proposed system in terms of a positive attitude (ATT), perceived usefulness (PU), perceived ease of use (PEOU), intention to use (ITU), trust (T), and the task–technology fit (TTF).

Many studies have examined users’ acceptance of health information technology [45,48]. However, their findings may not suit the context of older adults because the capabilities of this population are declining in terms of psychological aspects [49,50]. The present study preliminarily examined older adults’ perceived acceptance of the proposed gait and balance monitoring system augmented with sensor technology. Figure 2 shows that the participants perceived that using the system for gait and balance monitoring is a good idea (positive attitude: mean = 5.45, SD = 0.76) and easy (perceived ease of use: mean = 4.95, SD = 1.09), and that the system is useful in improving their health (perceived usefulness: mean = 5.32, SD = 0.83), is trustworthy (trust: mean = 5.04, SD = 0.88), and has a good fit between task and technology (good fit: mean = 4.97, SD = 0.84). The data for trustworthiness were relatively discrete. One possible reason is that it may take older adults more time to develop trust in gerontechnology, given the limited time for practicing using our smart devices. In general, the participants showed a positive intention to use the proposed system in their gait and balance management (intention to use: mean = 5.22, SD = 1.10).

Furthermore, we calculated the demographic correlation of acceptance perceptions (see Table 2). Logistic regression revealed that the participants with hypertension (odds ratio [OR] of 7.17, 95% confidence interval [95% CI] of [1.42, 36.06], *p* = 0.017), with no walking aids (OR of 8.66, 95% CI of [1.60, 46.8], *p* = 0.012), or with a higher MoCA score (OR of 1.28, 95% CI of [1.06, 1.54], *p* = 0.012) tended to believe that using the proposed system was useful for their health management. The participants with a fracture (OR of 13.04, 95% CI of [1.55, 109.86], *p* = 0.019), cataract (OR of 4.50, 95% CI of [1.14, 17.72], *p* = 0.031), no walking aids (OR of 5.86, 95% CI of [1.15, 29.93], *p* = 0.033), a higher ABC score (OR of 1.03, 95% CI of [1.00, 1.05], *p* = 0.037), or a higher MoCA score (OR of 1.23, 95% CI of [1.02, 1.47], *p* = 0.027) were more likely to believe that using the proposed system was easy. The participants with no walking aids (OR of 8.43, 95% CI of [1.37, 51.94], *p* = 0.022) or a higher MoCA score (OR of 1.25, 95% CI of [1.03, 1.53], *p* = 0.027) tended to trust the proposed system. The participants with a fracture (OR of 15.89, 95% CI of [2.00, 126.39], *p* = 0.009), a higher ABC score (OR of 1.04, 95% CI of [1.01, 1.06], *p* = 0.007), or a higher MoCA score (OR = 1.40, 95% CI [1.15, 1.71], *p* = 0.001) were more likely to believe that the fit between task and technology was good. No other significant demographic correlations were discovered (all the *p* values exceeded 0.05).

### 3.3. Sensor Data

The IMU (i.e., accelerometer and gyroscope) and depth camera (i.e., Kinect, Microsoft, Redmond, WA, USA) are cost effective with few constraints on the monitoring of movements [27,51,52]. In particular, a feature matrix containing important information on the frequency and intensity of motion extracted from the raw signals can be used together with statistical learning methods to predict the BBS and BESTest scores of older adults.

In the following, we use the examples of the 3M-TUG and FTSTS tests to elaborate the importance of device fusion, functional test fusion, and data fusion in the study of gait and balance. In the functional 3M-TUG test, we segmented the overall test into a postural transition (i.e., sit-to-stand and stand-to-sit) phase, a turning phase, and a walking (i.e., two 3-m intervals) phase using the IMU data [1,32,53]. The postural transition phase includes not only fundamental components of daily activities but also prerequisites for walking and standing (e.g., lower limb strength and joint range changes) [54,55,56]. The turning phase is an important indicator of balance confidence and walking limitations [57]. The walking phase allows us to identify older adults’ gait behavior and consequently gait patterns [58], involving stability, gait symmetry, and regularity [1].

**Figure 3:**
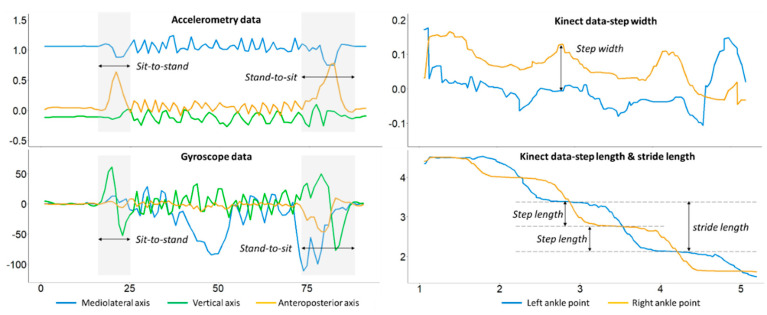
Segmentation of a 3M-TUG task using inertial sensor data and the measurements of step width and step length obtained from Kinect data (using the left and right ankles as two skeleton points).

Inertial sensors and the Kinect camera have their own unique functions. Figure 3 (left) presents the phase segmentation of a 3M-TUG test based on inertial sensor data, namely, accelerometer- and gyroscope-based data, capturing the different characteristics of body movements. Algorithms developed to segment the signal data into sit-to-stand, walking, and stand-to-sit phases can be found in other papers published by our team [1,11]. Figure 3 (right) presents measurements of the step width and step length taken using two Kinect skeleton key points, namely, the left and right ankles. The Kinect sensor-based step width was calculated using the differences between the two ankles, whereas the step length and stride length were calculated using the changes in distance to the Kinect camera [59]. In addition to the step length and stride length, the Kinect camera provides other important gait parameters, such as the gait speed, step time, stride time, ankle flexion, and knee adduction [60].

**Figure 4:**
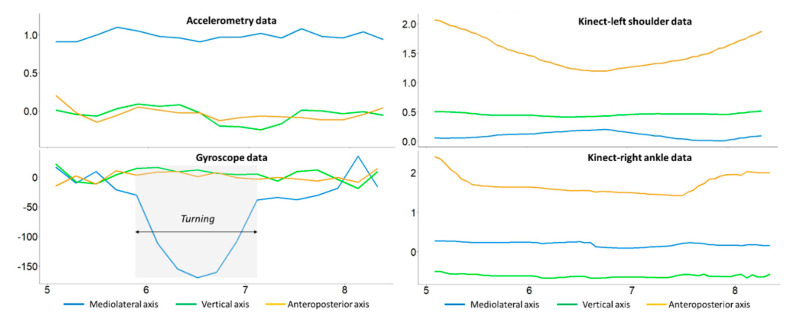
Comparison of the inertial sensor data and the Kinect data (using the left and right ankles as two skeleton points) for a 360-degree turning task.

Figure 4 presents the sensor data obtained using the inertial sensor (left) and Kinect camera (right) in the turning phases. In contrast with the above cases, there was an obvious turning phase in the gyroscope data, which could hardly be seen in the Kinect data (e.g., left shoulder data and right ankle data). Moreover, there were two possible data-related issues when individuals turned in front of the Kinect camera: (1) data could not be identified if the individual was too close to the Kinect camera and (2) data, such as those of the left and right shoulders, overlapped spatially when an individual turned his/her body (see Figure 4).

**Figure 5:**
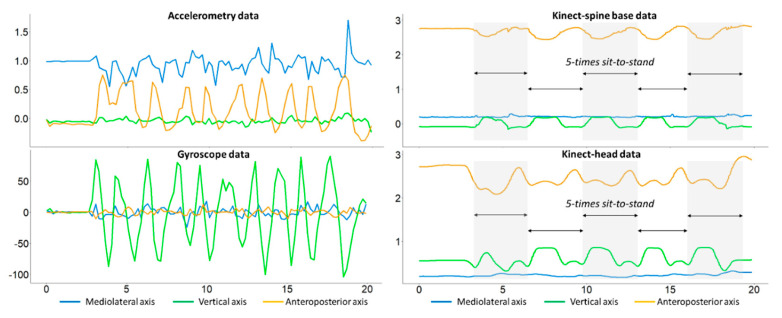
Comparison of the inertial sensor data and the Kinect data (using the left and right ankles as two skeleton points) for the FTSTS task.

Figure 5 presents the sensor data for the FTSTS task, showing that it is difficult to differentiate the sit-to-stand phase using inertial sensor data (left) if an individual makes more than one attempt to achieve this transition. In contrast, it can be clearly determined from the Kinect data (right) whether the individual finishes the transition by identifying the individual’s height. The integrated use of two devices is thus necessary, and the data obtained from the inertial sensor and Kinect camera are complementary in terms of obtaining meaningful results.

In fully exploiting the data available for gait and balance assessment, data fusion techniques at different levels of abstraction can be used to make inferences and improve accuracy. These techniques include signal-level fusion, pixel fusion, feature-level fusion, and symbol-level fusion. Statistical learning methods such as regression, and machine learning algorithms such as those of hidden Markov models can be used to correlate predictive variables with specific influencing factors. This approach can improve the clinical value of gait and balance assessments, allowing clinics to identify specific factors that increase fall risk and to design personalized interventions.

### 3.4. Implications and Limitations

The continuous monitoring of gait and balance is crucial for older adults, especially those who are prone to falling. The detection of any appreciable deterioration in gait and balance allows the application of timely and appropriate interventions intended to prevent further falls. The proposed system uses sensor technology and big data analytics to develop an innovative approach in the dynamic monitoring of gait and balance. In contrast with traditional gait and balance assessment methods, the proposed system can be used by caregivers at care centers, with minimal involvement from healthcare professionals (e.g., PTs). Additionally, the proposed system is expected to reduce the time required for assessment compared with traditional balance measures, and mitigate the burden of disease for an aging society in the long term.

In contrast to the majority of the existing literature that primarily employs uni-modal methodologies for gait and balance analysis, such as Kinect cameras [61] or IMUs [62], our newly proposed multi-sensor system for gait and balance assessment capitalizes on the concept of sensor fusion. This pioneering system integrates a variety of sensing devices that synergistically enhance the comprehensiveness and precision of gait and balance evaluations. For instance, during complex maneuvers such as rotations, where the Kinect camera may encounter obstructions due to overlapping body shadows, the data from IMUs can intervene to furnish valuable insight into the turning phase. This multi-sensor strategy not only circumvents the limitations of individual sensors but also cultivates multi-sourced data [63], enriching our dataset compared to the uni-sourced data employed in previous studies. The heterogeneous data procured from multiple sources augment our capacity for data analysis and algorithm development, thereby facilitating the construction of more refined and accurate models.

Moreover, while numerous studies concentrate on specific pathologies, such as neurological disorders [64] and glaucomatous individuals [65], our system distinguishes itself through its adaptability. The architecture of our system is engineered to accommodate a broad spectrum of clinical settings and diverse patient populations, thereby extending its applicability beyond specific patient groups. Last but not least, our system can be deployed either in a home-based continuous monitoring environment [66] or in a clinical environment [67]. It facilitates real-time feedback to healthcare providers remotely, enhancing patient care by allowing for prompt interventions and adjustments.

Theoretically, our proposed system is an example of a system that adopts big data analytics and sensor technology to solve the care problems of older adults. The proposed methods can extract quantitative features that are more meaningful than those extracted via conventional human-based assessment, to comprehensively reflect the gait and balance behaviors of older adults. Additionally, the extracted features can be used to improve the accuracy of other relevant systems. Furthermore, our research provides evidence about older adults’ acceptance of technology-augmented healthcare systems.

Practically, the proposed system could be disseminated to community-based centers, where older adults could routinely monitor their functional capability relating to falls with the aim of preventing avoidable hospitalizations. According to the warnings issued by the system, early interventions and optimal resource allocations could be conducted, which would reduce the costs incurred by the overall public healthcare system. The system was designed following human factor principles (e.g., being easy to implement), and caregivers at centers will thus be able to easily operate the system and perform assessments. Additionally, we will keep modifying the monitoring system (including all hardware and software) according to the findings of further usage in practice. A final easy-to-use package will be promoted to elderly care centers as a tailor-made system for the effective gait and balance monitoring of older adults in Hong Kong.

The present study has several limitations. First, the feasibility test was conducted on a one-time basis. In further implementing our system in practice, we need to conduct a longitudinal acceptance modeling study for the full version of our system to focus on factors that affect acceptance by older adults and healthcare professionals. Following that, targeted strategies (e.g., community-based technology support services and training workshops) could be promoted to improve the user acceptance of our proposed system. Second, strategies are needed to address safety risks in the use of the system. To minimize potential safety concerns associated with using the proposed system, we suggest that older adults perform a self-evaluation of their gait and balance in their affiliated community centers, under the supervision of center caregivers. Third, our sensor-technology-augmented system is designed to be a surrogate tool for gait and balance assessment, and not to provide a clinical diagnosis. Thus, when the system sends an alarm that the user has undergone statistically significant changes in their gait and balance behaviors, the user will be strongly advised to see their family doctor as soon as possible. Fourth, the individual’s gait and balance patterns should be further adjusted by incorporating other personal health-related information, such as the individual past medical history, drug prescriptions, lifestyle, and demographics (age and gender), as multiple variables.

## 4. Conclusions

The proposed system is novel in that it automatically analyzes sensor signals and presents gait and balance assessment results to older adult users and their families and caregivers via smart devices. Our preliminary studies led us to hypothesize that, by modeling the correlations between signal data and clinical balance scores, the proposed system will be translatable and thus facilitate the monitoring of gait and balance among community-dwelling older adults, and assist caregivers. Innovative healthcare solutions, such as telehealth, are a possible solution for supporting community caregivers in meeting the increasing health service demand. For older adults who usually find it more challenging to access or use rehabilitation programs or resources for gait and balance evaluation, our proposed system can be considered beneficial and consistent with their diverse abilities.

## Figures and Tables

**Table 1:** Demographic data of the 44 participants.

Numerical Variables	Mean (SD)	Median	Range
Age, years	78.5 (6.2)	78.0	68.0–88.0
Stature, cm	153.5 (7.3)	153.0	137.0–175.0
Body weight, kg	57 (10.8)	57.8	33.8–79.7
Body mass index, kg/m^2^	24.2 (4)	24.1	15.6–35.9
Health index (1–5)	3.7 (1.1)	4.0	1–5
MoCA (0–30)	22.9 (4)	23.0	12–29
ABC (0–100%)	54.5 (29.8)	61.3	0–96.9
BBS (0–56)	48.0 (7.1)	49.0	22–56
Brief-BESTest (0–24)	14.5 (4.5)	16.0	1–21
Categorical variables	Number, n (%)		
Female gender	38 (86.4%)		
Chronic disease			
Hypertension	33 (75.0%)		
Diabetes mellitus	8 (18.2%)		
Heart disease	9 (20.5%)		
Fracture	6 (13.6%)		
Arthritis	27 (61.4%)		
Cataract	26 (59.1%)		
Rheumatic pain	22 (50.0%)		
Fall history in the past 12 months	17 (38.6%)		
One fall	12 (27.3%)		
Two falls	3 (6.8%)		
Three falls	2 (4.5%)		
Walking assistance (Yes)	14 (31.8%)		

**Table 2:** Demographic correlations (OR; 95% CI) with participants’ perceptions of the system.

Demographic	Positive Attitude	Perceived Usefulness	Perceived Ease of Use	Intention to Use	Trust	Task–Technology Fit
Age	1.03 (0.92, 1.16)	1.1 (0.97, 1.24)	1.08 (0.96, 1.21)	1.05 (0.93, 1.18)	1.11 (0.97, 1.26)	0.95 (0.85, 1.07)
Male ^a^	3.99 (0.4, 40.15)	2.84 (0.29, 28.31)	2 (0.21, 18.76)	2.09 (0.23, 19.49)	0.92 (0.08, 10.33)	4.01 (0.41, 38.97)
Chronic disease ^b^						
Hypertension	0.76 (0.17, 3.55)	7.17 (1.42, 36.06) *	4.51 (0.96, 21.26)	2.08 (0.46, 9.39)	2.08 (0.39, 11.21)	1.76 (0.39, 7.92)
Diabetes mellitus	0.8 (0.16, 4.13)	0.67 (0.13, 3.5)	1.24 (0.25, 6.23)	0.58 (0.12, 2.88)	2.08 (0.34, 12.73)	0.57 (0.11, 2.88)
Heart disease	0.36 (0.08, 1.58)	0.33 (0.07, 1.45)	1.05 (0.25, 4.44)	0.42 (0.1, 1.79)	0.6 (0.12, 2.91)	0.59 (0.14, 2.51)
Fracture	0.46 (0.07, 3.25)	0.9 (0.13, 6.27)	13.04 (1.55, 109.86) *	0.74 (0.11, 5.01)	1.89 (0.24, 15.24)	15.89 (2, 126.39) **
Arthritis	2.49 (0.43, 14.48)	0.59 (0.1, 3.34)	0.27 (0.05, 1.55)	0.39 (0.07, 2.12)	0.71 (0.11, 4.48)	0.61 (0.11, 3.37)
Cataract	2.89 (0.74, 11.39)	1.9 (0.49, 7.37)	4.5 (1.14, 17.72) *	3.03 (0.79, 11.59)	1.28 (0.3, 5.43)	2.84 (0.74, 10.86)
Rheumatic pain	0.23 (0.04, 1.18)	0.69 (0.14, 3.38)	1.14 (0.24, 5.41)	1.07 (0.23, 5.05)	0.38 (0.07, 2.15)	1.36 (0.29, 6.49)
Falls ^c^	0.39 (0.08, 2.05)	1.81 (0.35, 9.36)	1.32 (0.26, 6.78)	0.59 (0.12, 2.92)	0.89 (0.15, 5.19)	0.38 (0.08, 1.95)
Walking aids ^d^	2.23 (0.45, 10.94)	8.66 (1.6, 46.8) *	5.86 (1.15, 29.93) *	2.5 (0.53, 11.74)	8.43 (1.37, 51.94) *	1.57 (0.33, 7.42)
ABC score	1.01 (0.99, 1.04)	1.02 (0.99, 1.05)	1.03 (1, 1.05) *	1.02 (0.99, 1.04)	1.02 (1, 1.05)	1.04 (1.01, 1.06) **
MoCA score	1.17 (0.97, 1.4)	1.28 (1.06, 1.54) *	1.23 (1.02, 1.47) *	1.1 (0.92, 1.31)	1.25 (1.03, 1.53) *	1.4 (1.15, 1.71) **
Health index	0.99 (0.49, 1.98)	0.83 (0.42, 1.67)	0.95 (0.48, 1.88)	1.14 (0.58, 2.24)	0.67 (0.31, 1.47)	1 (0.5, 1.98)
Stature	0.68 (0.3, 1.54)	0.7 (0.31, 1.59)	0.51 (0.22, 1.15)	1.02 (0.46, 2.24)	0.62 (0.26, 1.48)	0.87 (0.39, 1.93)
Body weight	1.62 (0.55, 4.77)	1.52 (0.52, 4.49)	2.29 (0.78, 6.72)	0.99 (0.35, 2.83)	1.92 (0.61, 6.04)	1.17 (0.41, 3.36)
Body mass index	0.34 (0.03, 3.75)	0.39 (0.03, 4.38)	0.16 (0.02, 1.8)	0.95 (0.09, 9.83)	0.27 (0.02, 3.4)	0.85 (0.08, 8.89)

Male ^a^: data for female participants are treated as the reference (OR of 1); Chronic disease ^b^: data for specific diseases are treated as the reference (OR of 1); Falls ^c^: data for fallers are treated as the reference (OR of 1); Walking aids ^d^: data for walking-aid users are treated as the reference (OR of 1); * *p* < 0.05, ** *p* < 0.01.

## Data Availability

The datasets generated and/or analyzed during the current study are not publicly available due to Institutional Review Board-related matters but are available from the corresponding author on reasonable request.

## Abbreviations

SD	Standard deviation
3M-TUG	3-m timed up and go
FTSTS	Five times sit o stand
BBS	Berg balance scale
IMU	Inertial measurement unit
AI	Artificial intelligence
PTs	Physiotherapists
BESTest	Balance evaluation systems test
ABC	Activities-specific balance confidence
MoCA	Montreal cognitive assessment
ATT	Attitude
PU	Perceived usefulness
PEOU	Perceived ease of use
ITU	Intention to use
T	Trust
TTF	Task–technology fit
OR	Odds ratio
